# Pedigree reconstruction and genetic analysis of major ornamental characters of ornamental crabapple (*Malus* spp.) based on paternity analysis

**DOI:** 10.1038/s41598-022-18352-z

**Published:** 2022-08-18

**Authors:** Hao Rong, Bin Huang, Xin Han, Kai Wu, Meng Xu, Wangxiang Zhang, Feng Yang, Li-an Xu

**Affiliations:** 1 College of Forestry, Nanjing Forestry University, Nanjing, China; 2 Co-Innovation Center for Sustainable Forestry in Southern China, Nanjing Forestry University, Nanjing, China; 3 Jiangxi Provincial Key Laboratory of Camellia Germplasm Conservation and Utilization, Jiangxi Academy of Forestry, Nanchang, China; 4 Qingdao Municipal Supervision Consulting CO., LTD, Qingdao, China

**Keywords:** Plant sciences, Plant breeding, Plant genetics

## Abstract

Ornamental crabapple is an important woody ornamental plant in the Northern Hemisphere. Its flowers, fruits, leaves and tree habit are all important ornamental characters. As there has been no research on the selection of superior parents and phenotypic variation, new varieties of ornamental crabapple are mainly selected from open-pollination progeny. In order to explore the transmission rule of ornamental traits between parents and offspring of crabapple, and to provide a basis for the selection of hybrid parents for directional breeding, 14 pairs of SSR markers were used in this study for paternity analysis of 384 offspring from 4 female parents crossed with 91 candidate male parents. And 273 offspring (71.1%) were matched with only the father at a 95% strict confidence level. We reconstructed 7 full-sib families (number of progeny ≥ 10) on the basis of the paternity analysis results. Genetic analysis of characters in the full-sib families revealed that green leaves and white flowers were dominant traits. All the hybrid offspring from the white flower (♀) × non-white flower (♂) cross produced white flowers, while 7.04% produced non-white flowers when both parents had white flowers. The results showed that white flowers might be a dominant qualitative trait in crabapple, while the depth of red was a quantitative trait. The genetic characteristics of green and non-green leaves and the depth of red of the peel were similar to flower color. Compared with the upright and spreading traits, the weeping trait was recessive. Some progeny showed an earlier blooming period, indicating the possibility of breeding for blooming period. Our findings are important for parent screening and improving the breeding efficiency of new varieties in ornamental crabapple hybridization.

## Introduction

Ornamental crabapples (*Malus* spp.) are plants in the Rosaceae with a fruit diameter of less than 5 cm and flowers, fruits, leaves and other traits of ornamental value. As important woody ornamental plants in temperate regions of the Northern Hemisphere, they are widely used in landscaping and landscape design^1,2^. There are many varieties of ornamental crabapples. Among the more than 200 varieties reported at present, only slightly more than 60 have known parents^2^. Most of the varieties were selected from the progeny of natural hybrids, with complex genetic backgrounds and unclear genetic relationships. The breeding of crabapple varieties is still achieved by selecting the offspring of natural hybrids on the basis of excellent ornamental traits and retaining them through vegetative propagation. For example, the new cultivars *M*. ‘FengHong NiChang’^3^ and *M*. ‘Fen Balei’^4^ were selected from *Malus halliana* and *Malus micromalus*, respectively. As it is difficult to identify the male parent in this process, it is impossible to breed a large number of new varieties with high efficiency by artificial crossbreeding for traits, which restricts the directed breeding of ornamental crabapples. Our laboratory aimed to use a variety of traditional methods to conduct artificial hybridization of ornamental crabapples. Due to the low fertility of different varieties and the self-incompatibility of gametophytes in *Malus* spp., the success rate was very low, and it was difficult to obtain enough hybrid offspring for effective selection, which limited the efficient advancement of artificial hybridization for ornamental crabapples.

El-Kassaby et al. (2009) presented a strategy for forest breeding called ‘breeding without breeding’ (BWB). This method did not require any controlled pollination or experimental field testing, which are considered to be the most resource-consuming steps in breeding. The method involved using paternity analysis to create a full-sib family and performing quantitative genetics analyses for further genetic improvement or selecting the parents in cross-breeding which could greatly improve breeding efficiency^5–7^. The key step in BWB was to use parentage analysis to reconstruct the family, that is, to use genetic markers to trace the male parent of half-sibling families. The markers used for paternity analysis mainly include random amplification polymorphic DNA (RAPD)^8^, amplified fragment length polymorphisms (AFLPs)^9^, simple sequence repeats (SSRs)^10^, and single nucleotide polymorphisms (SNPs)^11^. With the development of molecular marker technology, the improvement of analysis methods and the development of analysis software, the efficiency and accuracy of paternity analysis have been improved^12,13^. Among these markers, SSRs are short tandem repeat sequences with 1–6 nucleotides as the repeat unit. They are commonly used in paternity analysis because they are widely distributed throughout the genome of eukaryotes, with the advantages of codominance, high polymorphism, and high stability.

The BWB strategy provides a new approach for improving the current breeding methods of ornamental crabapples. In this study, we surveyed the traits of existing half-sib families and used SSR molecular markers for parentage analysis. Then, we analyzed the crossbreeding compatibility between varieties, the genetic variation of the traits between parents and offspring, and the efficiency of different hybrid combinations to produce qualified ornamental characters. This study is expected to provide a basis for parental selection of artificial hybrids in directional breeding of new varieties of ornamental crabapples.

## Methods

### Plant materials

The materials used in the experiment were obtained from the national repository of *Malus* spp. Germplasm (Nanjing Forestry University), which is located in Jiangdu District, Yangzhou City, Jiangsu Province (119°55′E, 32°42′N). There were 105 kinds of crabapple cultivars collected domestically and internationally, all of them comply with relevant institutional, national, and international guidelines and legislation, and there is no intellectual property issue. Thirty clones of each variety were planted in a 2 × 3 m plot, and all of them were between five and eight years old, i.e., in the full bloom phase. In Fall 2013, seeds of *M.* ‘Sweet Sugartyme’, *M.* ‘Darwin’, *M.* ‘Red Sentinel’, and *M.* ‘Rainbow’ were collected from the germplasm. The male parent of these materials was unknown and might be one of 91 cultivars in the nursery with overlapping flowering (Table S1 in Supplementary Information 1). A total of 221, 450, 218, and 206 offspring were obtained from four half-sibling families in 2019, and 96 individuals were randomly selected from each family. The young leaves of 384 offspring and 91 candidate parents were collected, placed in iceboxes, and quickly transferred to the laboratory for preservation in a − 80 °C freezer in 2019.

### Trait investigation and statistics

Ten traits were investigated to analyze the degree of trait separation and variation between parents and offspring in 2019. The methods of investigation and classification were as described by Liu^14^ (Table 1).

**Table 1:** Ornamental trait classification.

Characters	classification					
	0	1	2	3	4	5
Blooming period	None	Early	Medium	Late	–	–
Flower color	–	White	Pink	Red	Light purple	Purple
Leaf color	–	Green	red	Purple	–	–
Leaf shape		Ovate-round	Broadly ovate	Obovate	Broadly elliptical	Long elliptical
Leaf surface	Sparse	Medium	Thick	–	–	–
Fruit color	–	Green	Yellow	Red	–	–
Fruit size	–	Very small (<6mm)	Small (6–13mm)	Medium (13–25mm)	Large (25–50mm)	–
Fruit calyx	–	Absent	Always present	–	–	–
Glossiness fo skin	–	Strongly expressed	Weakly expressed	Absent	–	–
Tree habit	–	Upright (<30°)	Spreading (30°–70°)	Drooping (70°–90°)	Weeping (>90°)	–

The varieties with an early blooming period bloomed between March 31 and April 4, and those with a medium blooming period bloomed between April 5 and April 9. Varieties that entered the blooming period after April 10 were recorded as having a late blooming period, and those that still had not bloomed in May were considered non-blooming These categories were based on the blooming period of *M*. ‘Pink Spires’ (March 31), which began blooming earliest. The color of flowers, leaves and fruit was checked on the sunny side using the Royal Horticultural Society Standard Color Chart (RHSCC). The leaf base was wide, the length was approximately twice the width (ovate-round), and the length and width were similar to those of broadly ovate leaves. In contrast to ovate-round leaves, the narrow leaf base led to an obovate shape. When the middle part of the leaf is the widest and the length is approximately 1.5–2 times the width, the leaf is recorded as broadly elliptical; when the length is 3 to 4 times the leaf width, the leaf is recorded as long elliptical.

### DNA extraction

Total genomic DNA was extracted using BioTeke Rapid Plant DNA Extraction Kit (BioTeke, Beijing, China). DNA concentrations were estimated with a NanoDrop 2000 spectrophotometer (NanoDrop Technologies, Wilmington, DE, USA), and the qualified DNA was normalized to a concentration of 50 ng/μl for polymerase chain reaction (PCR).

### Genotyping with SSR markers

Fourteen pairs of SSR primers were used in this experiment, 11 were from previous studies and the other 3 were developed by our laboratory based on transcriptome data (Table 2). PCR amplification with all primers was carried out using an ABI Veriti 96 PCR system (Thermo Fisher Scientific, MA, USA). The reaction mixtures of 15 μl contained 1 × buffer, 6 mg/l genomic DNA, 0.25 μmol/L each SSR forward and reverse primer, 0.25 mmol/L dNTPs, 2 mol/L MgCl2 and 1.25 U of Taq polymerase, all of which were obtained from Takara (Takara Biomedical Technology, Dalian, Co., Ltd.). The PCR system was adjusted according to Wang et al*.*^15^ and the program involved an initial denaturation step of 4 min at 94 °C, followed by 32 cycles at 94 °C for 45 s, the appropriate annealing temperature for 30 s, 72 °C for 40 s, and an extension cycle of 1 min at 72 °C. The PCR products were separated on an ABI 3730XL instrument (Thermo Fisher Scientific, MA, USA).

**Table 2:** SSR-PCR primers.

Code	Sequence	Tm	PIC
GD142^16^	GGCACCCAAGCCCCTAA	62	0.828
	GGAACCTACGACAGCAAAGTTACA		
CH03d07^17^	CAAATCAATGCAAAACTGTCA	60	0.858
	CGCTTCTGGCCATGATTTTA		
GD96^16^	CGGCGGAAAGCAATCACCT	60	0.864
	GCCAGCCCTCTATGGTTCCAGA		
MES2^18^	CACCACAACCCAAAGCAA	60	0.695
	GAGCAAAGCATCCAGCAA		
gssr-11*	GTAACTTGGAAGGGGAAGGG	60	0.859
	TCGACCATACAAATTGCTGC		
Hi02f06^19^	TAAATACGAGTGCCTCGGTG	62	0.877
	GCAGTTGAAGCTGGGATTG		
gssr-2*	TCGTGTGAGAGATGAAACCG	52	0.898
	GGCCATTAGCTCCACATCAT		
gssr-21*	AGGGAATGACGTTCCAACTG	62	0.679
	ATGATCAAAGCCCATGGAAG		
Hi02c07^19^	AGAGCTACGGGGATCCAAAT	59	0.864
	GTTTAAGCATCCCGATTGAAAGG		
CN444794-SSR^19^	CATGGCAGGTGCTAAACTTG	56	0.877
	GTTTGCAACTCACACAATGCAAC		
CH01f09^20^	ATGTACATCAAAGTGTGGATTG	56	0.784
	GGCGCTTTCCAACACATC		
CH01h10^17^	TGCAAAGATAGGTAGATATATGCCA	62	0.870
	AGGAGGGATTGTTTGTGCAC		
CH04b10^17^	AGCAGACCAACGCATATCAA	62	0.851
	TAATCTGTGCCGGTATGTGC		
CH05g03^17^	GCTTTGAATGGATACAGGAACC	60	0.818
	CCTGTCTCATGGCATTGTTG		

*Indicates an EST-SSR primer developed by our laboratory based on the transcriptome data.

### Data analysis

Genotyping results were analyzed by Peak Scanner V1.0 software (Thermo Fisher Scientific, MA, USA). The segments were arranged in order from smallest to largest as A, B, C…, the segments with the same base size were represented by the same capital letter, and missing data were indicated by ‘..’. The number of observed alleles (Na), observed heterozygosity (Ho), and Nei’s diversity index was determined using POPGENE version 1.32 software^21^. Paternity analysis was performed based on the maximum likelihood method by comparing genotypes of known maternal origin and offspring against candidate paternal materials using Cervus 3.0 software^22^ with a genotyping error rate set to 0.01.

## Results

### Genetic diversity

A total of 216 alleles were obtained for the 14 pairs of SSR primers based on 91 candidate parents. The number of alleles per SSR locus ranged from 8 to 24, with an average of 15.4 alleles per locus. In general, the average Ne and He were 6.10 and 0.81, respectively. In addition, 180 alleles were obtained based on 384 offspring. The number of alleles per SSR locus varied from 7 to 20, with an average of 12.9, and the average Ne and He were 4.87 and 0.76, respectively (Table 3). Since half of the genetic variation of offspring comes from only 4 mothers and half comes from the part of the 91 candidate parents. Although there were far more offspring than candidate parents, the genetic diversity of the progeny was still lower than that of the candidate parents (except for gssr-21). All the genotypes from the 14 pairs of SSR primers were available in Supplementary Information 2.

**Table 3:** Genetic diversity indicators of candidate parents and offspring at 14 loci.

Locus	Na	Ne	He
	Parents/Offspring	Parents/Offspring	Parents/Offspring
GD142	14/14	5.60/5.46	0.83/0.82
CH03d07	20/15	6.46/3.92	0.85/0.75
GD96	17/16	7.05/6.46	0.86/0.85
MES2	12/7	3.74/2.74	0.72/0.62
gssr-11	17/17	6.76/5.87	0.83/0.83
Hi02f06	14/14	6.40/6.10	0.85/0.84
gssr-2	24/20	9.68/6.53	0.9/0.85
gssr-21	12/13	3.68/2.53	0.73/0.61
Hi02c07	17/13	6.98/6.55	0.86/0.85
CN444794-SSR	8/7	4.01/3.34	0.61/0.48
CH01f09	12/8	3.29/3.83	0.70/0.74
CH01h10	17/13	9.94/4.20	0.90/0.76
CH04b10	17/13	5.62/4.93	0.83/0.80
CH05g03	15/10	6.23/5.77	0.84/0.83
Mean	15.43/12.89	6.10/4.87	0.81/0.76
St.Dev	3.79/3.59	1.97/1.38	0.08/0.11

Na: number of alleles, Ne: effective number of alleles, He: expected heterozygosity.

### Paternity analysis of half-sib families

**Figure 1:**
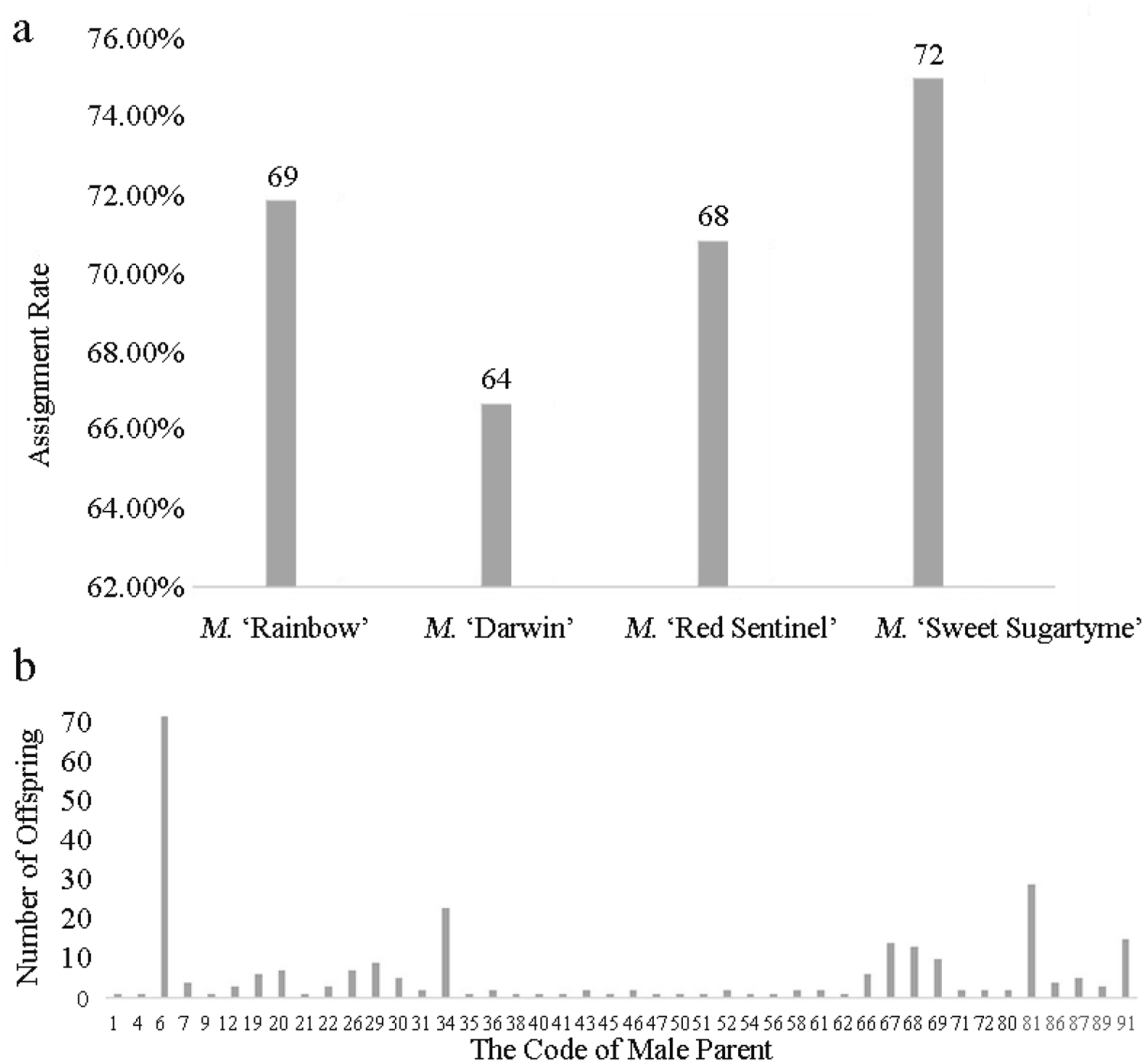
(**a**) The number and assignment rate of the 4 half-sib families by paternity analysis. (**b**) The code of 44 male parents and the number of offspring they produced.

Of the 384 offspring from the 4 half-sib families, 273 (71.09%) were matched to a unique paternal tree at a 95% strict confidence level. The average matching success rate of the 4 families was 71.09%, with the *M.* ‘Sweet Sugartyme’ family being the highest at 75% and the *M.* ‘Darwin’ family being the lowest at 66.67% (Fig. 1a). Table S2 in Supplementary Information 1 shows the correspondence of paternal materials and offspring at a 95% strict confidence level. Only 44 of the 91 candidate parents produced progeny, and the number of progeny produced by the 44 male parents ranged from 1 to 72. In addition, as the male, *M.* ‘Red Sentinel’ (No. 6) produced 72 progeny with other ornamental crabapples, showing the highest reproductive contribution rate. *M.* ‘Winter Red’ and *M.* ‘Sweet Sugartyme’ ranked second and third, producing 29 and 23 offspring, respectively. Fourteen, 13 and 3 progenies were produced by *M.* ‘Weeping Madonna’ (67), *M.* ‘Louisa Contort’ (68) and *M.* ‘Rainbow’ (12), respectively. *M.* ‘Darwin’ (25) did not produce offspring when used as the father. In addition, 15 paternal parents produced only one offspring (Fig. 1b).

### Family reconstruction and trait variation analysis of the full-sib families

According to the paternity analysis, we reconstructed the full-sib families of ornamental crabapples and selected 7 hybrid combinations with more than 10 progenies from these families (Table S3 in Supplementary Information 1). The characters of 7 parents are shown in Table 4.

**Table 4:** The characters of parents.

	‘Rainbow’	‘Darwin’	‘Red Sentinel’	‘Sweet Sugartyme’	‘Winter Red’	‘Weeping Madonna’	‘Louisa Contort’
Blooming period	1	1	2	2	2	2	1
Flower color	1	2	1	1	1	1	2
Leaf color	1	2	1	1	1	1	1
Leaf shape	4	4	4	4	5	2	4
Leaf surface	1	1	2	1	1	1	2
Tree habit	1	2	2	1	2	4	3
Fruit size	3	3	2	3	2	3	3
Fruit color	3	2	2	3	2	2	1
Fruit calyx	1	1	2	1	1	2	1
Glossiness of skin	2	1	1	2	1	2	2

*M.* ‘Darwin’ was only a female parent. *M*. ‘Red Sentinel’, *M.* ‘Sweet Sugartyme’ and *M*. ‘Rainbow’ could be both the female parent and the male parent. *M*. ‘Winter Red’, *M*. ‘Weeping Madonna’ and *M*. ‘Louisa Contort’ were only male parents.

A summary of the results from flower color, leaf color, fruit color, leaf shape, fruit size and tree habit is shown in Table 5. When the flower color was divided into white and non-white (pink and red), white was dominant over non-white. The hybrid progenies of white flower (♀) × non-white flower (♂) crosses all had white flowers. When both parents had white flowers, 92.92% of the offspring had white flowers and 7.04% had non-white flowers, indicating that the production of white flowers was not recessive. The offspring with green leaves accounted for 90.76%, and only 4 red and 6 purple progenies resulted from red leaf (♀) × green leaf (♂) crosses. There were only 2 red-leaved individuals among the hybrids, and both parents had green leaves. These results prove that green leaves were dominant over red leaves. The ratio of red to non-red (yellow and green) offspring was near 1:1 in red fruit (♀) × non-red fruit (♂) crosses. The red-fruited offspring accounted for 80% when a red-fruited female was crossed with a green-fruited male. The other two combinations mainly produced non-red and red peels. Overall, the ratio of red to non-red fruits was close to 1:1, and red and yellow were the main fruit colors, accounting for 45.38% and 40.00%, respectively.

**Table 5:** Distribution of offspring for hybrid combinations with different traits.

Characters	Female	Male	Classification					
			0	1	2	3	4	5
Flower color	1	1		66	4	1	0	0
	2	1		25	24	0	0	0
	1	2		10	0	0	0	0
Leaf color	1	1		79	2	0		
	2	1		39	4	6		
Fruit color	3	2		8	24	27		
	2	2		5	22	22		
	2	3		5	5	2		
	3	1		1	1	8		
Leaf shape	4	4	0	0	2	0	78	14
	4	5	0	0	0	0	15	10
	4	2	0	0	1	0	10	0
Fruit size	3	2		3	35	59	0	
	2	3		0	5	7	0	
	3	3		0	0	21	0	
Tree habit	1	2		5	39	4	0	
	2	2		3	38	7	1	
	2	1		1	10	1	0	
	1	4		1	8	1	1	
	1	3		0	7	2	1	

Spreading was the most common habit among the offspring. Differentiation occurred in the progeny regardless of parental tree habit. For example, most of the offspring were spreading and a few offspring were drooping when an upright female was crossed with a spreading male. Approximately 2% of progenies were weeping when two spreading parents were crossed. However, the proportion of spreading offspring (approximately 10%) was significantly increased if the male or female parent was drooping or weeping.

Parents had similar effects on the leaf shape and fruit size of offspring in the7 full-sib families. It was rare that these two traits in offspring deviated significantly from those of the parents.

Table 6 shows the results for four traits, including leaf surface, glossiness of skin, fruit calyx, and blooming period. The leaf surface of offspring was affected by the parents; most progenies were similar to the parents, but a few were differentiated (transgressive inheritance). There were two types of strongly expressed and weakly expressed glossiness of skin in parents. The phenotypes of offspring were mostly within the range of values observed for the parents, but some progenies did not display one of the parents’ phenotypes.

**Table 6:** Distribution of offspring for hybrid combinations with different traits.

Characters	Female	Male	Classification					
			0	1	2	3	4	5
Leaf surface	1	2	3	37	42			
	1	1	6	11	19			
	2	1	0	10	2			
Glossiness of skin	2	1		24	13	11		
	1	1		37	10	2		
	1	2		1	8	3		
	2	2		6	5	10		
Fruit calyx	1	2		50	33			
	1	1		23	12			
	2	1		10	2			
Blooming period	1	2	0	22	65	0		
	2	2	0	3	30	0		
	2	1	0	1	9	0		

Only two types of blooming periods were observed for the parents and offspring, early and medium, with 80.00% of progeny showing the medium type. Similarly, the fruit calyx trait had only two states: absent and always present. The fruit calyx was absent in 63.85% of progenies, regardless of parental phenotype.

## Discussion

Genetic diversity is not only an important index used to measure species’ ability to adapt to changing environments but also a key factor affecting plant genetic improvement^23^. Studying the genetic diversity of ornamental crabapple varieties provides a molecular basis for hybridization selection. Hokanson et al.^24^ used 8 pairs of SSR primers to analyze the genetic diversity of 142 *Malus* plants, and the average He was 0.623. Kumar et al.^25^ used SSR molecular markers to analyze the genetic diversity of wild crabapple populations in the Himalayan region of India, and the He value was 0.506. In this study, the average He of 91 ornamental crabapple varieties was 0.81, which was significantly higher than the previously reported values. On the one hand, the 91 parental varieties in this study were from a wide range of sources, and compared with that of wild populations, their genetic background was complex, so the diversity level was higher. On the other hand, the number of markers used in this study was higher than that used in previous studies, and the capillary electrophoresis technology was more accurate. Moreover, the 384 progenies of the 4 families also had a high level of genetic diversity (He = 0.76), but the level was lower than that of the candidate parents (0.81). The main reason was that not all candidate parents provided pollen, and the paternal parents of these progeny came from only 44 varieties.

Paternity analysis has been extensively used in various plant studies, such as paternity testing, pedigree reconstruction, mating system examination and dynamic changes in genetic diversity in generations. Ai et al.^26^ used 11 pairs of SSR primers to analyze 286 seeds from a *Pinus massoniana* seed orchard with 129 candidate paternal clones, and paternity at a 95% and an 80% confidence level was determined for 25 seeds (8.80%) and 107 seeds (37%), respectively. However, in a small candidate parent population of *Moringa oleifera* with only 60 male parents, 8 pairs of SSR primers assigned fathers for 155 of 288 seeds (53.82%) based on a 95% strict confidence level^27^. By comparing the results of the two studies, it can be seen that the number of candidate parents^28^ and polymorphism of the SSR molecular markers used are the main factors that affect the accuracy and efficiency of paternity analysis. Furthermore, the sampling intensity of candidates is another important factor in paternity analysis. In this study, 91 candidate paternal parents were screened through phenological observations. Using 14 pairs of SSR primers at a 95% confidence level, 273 offspring (71.1%) were matched. It was obvious that using capillary electrophoresis to replace polyacrylamide gel electrophoresis was also an important way to improve efficiency and accuracy. Further analysis of the distance between the males and females in the nursery showed that *M.* ‘Red Sentinel’ and *M.* ‘Winter Red’, the male parents that produced the most offspring, were located far from the female parents, indicating no obvious relationship between pollination success rate and the distance of pollen transmission. The compatibility of male and female gametes might be the key factor affecting the pollination success rate. *M.* ‘Red Sentinel’ and *M.* ‘Winter Red’ have good compatibility with most varieties when used as male or female parent and are suitable as parents for hybrid experiments.

In this study, the genetic characteristics of traits in parents and offspring were analyzed through reconstructed full-sib families to provide a basis for parental selection in crossbreeding. Flower color is one of the main ornamental traits of crabapple, but there are no reports on the genetics of this trait in ornamental crabapple. According to genetic studies on the flower color of *Camellia azalea* Wei and *Hibiscus coccineus* Walter, white flowers are recessive to red flowers^29,30^. However, Han et al.^31^ found that the petal color of F_1_ offspring derive from the cross between cabbage with yellow petals (♀) and Chinese kale with white petals (♂) was white, and the segregation conformed to a Mendelian ratio of 3:1 in F_2_ offspring originating from self-pollination of F_1_ plants, proving that white petals were dominant over yellow petals. In the natural population of ornamental crabapples examined in this study, the flowers were mostly white. If flower color was divided into white and non-white (pink and red), the offspring from the cross between parents with white flowers (♀) and non-white flowers (♂) all had white flowers, indicating that white flowers were dominant over non-white flowers. When both parents had white flowers, only 7.04% of the offspring had non-white flowers, which again indicated that the white flower trait was dominant. Of course, the results need to be confirmed by crossing two parents with red flowers. In addition, the intensity of red was a continuous trait showing quantitative characteristics, and the molecular mechanism underlying its formation needs to be further studied.

The accumulation of anthocyanins causes red leaves in many crops and ornamental plants. Previous studies proved that the inheritance of red or purple leaves followed a monogenic recessive pattern^32,33^. In contrast, red or purple leaves are controlled by a single dominant gene in birch, copper beech and *Brassica juncea*^34–36^. Huang et al.^37^ found that leaf color was determined by a single locus and that the purple leaf phenotype was recessive to the green leaf phenotype by selfing *Kalanchoe garambiensis* (purple leaves) and *K. garambiensis* G. (green leaves). Our study showed that 97.53% of the offspring resulting from crosses between parents with green leaves also produced green leaves. The green leaf trait was observed among 79.59% of offspring from red (♀) × green (♂) crosses. This finding suggests that green leaves were dominant over red or purple leaves. However, the segregation ratio of green and red leaves in offspring deviated from that expected under Mendelian inheritance, which might be related to the quantity of offspring.

Previous studies on apples found that fruit color was composed of background and surface color. Red was dominant over yellow and controlled by the single gene *Rf*^38–40^. However, some scholars believe that the inheritance of skin color is regulated by major genes as well as polygenes. Although red is dominant, the intensity of red is affected by polygenes. It is a qualitative trait with a quantitatively inherited character^41^. In our study, the progenies with red fruits accounted for 80% of the progenies obtained from red (♀) × green (♂) crosses, and the segregation ratio of red and yellow was close to 1:1 in some full-sib families. We speculated that red peel was also dominant in crabapple.

Tree habit is a major ornamental trait controlled by both major genes and polygenes^42^. It has been proven that the weeping trait is regulated by single recessive genes in peach and *Canadian redbud*^33,43^. There were not only major *pl* genes but also some polygenes involved in controlling the weeping trait in *Prunus mume*^44^. In our data, upright and spreading were the main tree habits in offspring, and progenies rarely exhibited drooping and weeping. A few drooping and weeping progenies appeared in the crosses of upright (♀) × spreading (♂) and spreading (♀) × spreading (♂), respectively. The proportion of drooping and weeping in offspring was significantly increased if a parent was drooping or weeping. The results showed that weeping and drooping were recessive. However, the segregation ratio of two full-sib families’ offspring deviated from that expected under Mendelian inheritance, and we speculated that the production of weeping branches in crabapple may be similar to that in *P. mume*. Dougherty et al.^45^ revealed four genomic regions, W, W2, W3 and W4, that were significantly associated with weeping by performing genetic mapping in the F_1_ generation of a cross between *Malus* ‘Cheal’s Weeping’ and *Malus* ‘Evereste’, and W was the major locus, which further supported the inference of this study. We should select a parent with drooping or weeping branches for crossbreeding if we want to obtain more offspring with weeping branches.

The blooming period is an important trait in ornamental plants. It is valuable because earlier or later blooming periods will extend the viewing period of the species. Shen et al.^46^ found that the blooming period of progeny from the cross between *Plumbago auriculata* and *P. auriculata* f. alba was much earlier than that of the parents, which showed obvious heterosis. Furthermore, the authors proved that the blooming period was controlled by two pairs of major genes with additive dominance, with dominant effects predominating. In our study, the parents had early or medium blooming periods, and most progenies showed medium blooming periods. However, three progenies resulting from the cross between two medium-blooming parents showed early blooming. This result indicated the possibility of crossbreeding ornamental crabapple during the blooming period, although there were no later flowering plants among the existing hybrid combinations.

## Conclusion

At present, the method used to breed new varieties of ornamental crabapple mainly involves open-pollination offspring. This approach is inefficient and depends on the abundance of male parents, as well as the physical distance between the parents. Over time, efficiency will gradually decline and be difficult to sustain. By paternity analysis, we found that *M*. ‘Red Sentinel’ and *M*. ‘Winter Red’ were suitable as parents for hybrid experiments. The green leaves and white flowers were dominant traits, and they might be a dominant qualitative trait in crabapple. The weeping trait was rare and recessive compared with the upright and spreading traits. Interestingly, some progeny had an earlier blooming period than their parents, which indicated the possibility of changing the blooming period by cross-breeding. According to our results, we identified hybrid combinations with a high success rate, plentiful progeny variation and an increased possibility of producing ornamental varieties for artificial hybridization, which will improve the efficiency of new variety breeding.

## Supplementary Information

Supplementary Information 1.

Supplementary Information 2.

## Data Availability

Development SSR primer pairs have been deposited to GenBank, accession numbers are ON402244, ON402245 and ON402246.

## References

[1] Knudsen JT, Tollsten L, Bergström LG (1993). Floral scents—a checklist of volatile compounds isolated by head-space techniques. Phytochemistry.

[2] Zhou T (2020). A binary-based matrix model for *Malus corolla* symmetry and its variational significance. Front. Plant Sci..

[3] Fan J (2019). ‘Fenghong nichang’ flowering crabapple. HortScience.

[4] Zhou T (2019). ‘Fen balei’ crabapple. HortScience.

[5] El-Kassaby YA, Lstibůrek M (2009). Breeding without breeding. Genet. Res..

[6] El-Kassaby YA, Cappa EP, Liewlaksaneeyanawin C, Klápště J, Lstibůrek M (2011). Breeding without breeding: Is a complete pedigree necessary for efficient breeding?. PLoS ONE.

[7] Wang X-R, Torimaru T, Lindgren D, Fries A (2009). Marker-based parentage analysis facilitates low input ‘breeding without breeding’ strategies for forest trees. Tree Genet. Genomes.

[8] Harada T, Matsukawa K, Sato T, Ishikawa R, Saito KM (1992). DNA-RAPDs detect genetic variation and paternity in Malus. Euphytica.

[9] Zhao T, Shen H, Yao Y, Cao Q, Song T (2010). Identification of parentage of ornamental crabapple seedlings using AFLP markers. Acta Hortic. Sinica.

[10] Bowers J (1999). Historical genetics: The parentage of chardonnay, gamay, and other wine grapes of Northeastern France. Science.

[11] Muranty H (2020). Using whole-genome SNP data to reconstruct a large multi-generation pedigree in apple germplasm. Bmc Plant Biol..

[12] Weir BS, Anderson AD, Hepler AB (2006). Genetic relatedness analysis: Modern data and new challenges. Nat. Rev. Genet..

[13] Blouin MS (2003). DNA-based methods for pedigree reconstruction and kinship analysis in natural populations. Trends Ecol. Evol..

[14] Liu, Y. N. Studies of sandard description and database construction of malus cultivars. *Chin. Acad. For.* (2018).

[15] Wang LX, Zhang XJ, Shi XY, Gao H, Zhao ZY (2012). Establishment of SSR fingerprinting database on major apple (*Malus* × *domestica*)cultivars. J. Fruit Sci..

[16] Hemmat M, Weeden NF, Brown SK (2003). Mapping and evaluation of malus ×domestica microsatellites in apple and pear. J. Am. Soc. Hortic. Sci..

[17] Liebhard R (2002). Development and characterisation of 140 new microsatellites in apple (Malus x domestica Borkh.). Mol. Breed..

[18] Yao L (2010). Exploitation of Malus EST-SSRs and the utility in evaluation of genetic diversity in Malus and Pyrus. Genet. Resour. Crop Evol..

[19] Silfverberg-Dilworth E (2006). Microsatellite markers spanning the apple (Malus x domestica Borkh.) genome. Tree Genet. Genomes.

[20] Gianfranceschi L, Seglias N, Tarchini R, Komjanc M, Gessler C (1998). Simple sequence repeats for the genetic analysis of apple. Theor. Appl. Genet..

[21] Yeh, F.-C., Yang. R.-C. & Boyle, T.B.J. POPGENE Version 1.32 Microsoft Windows-based freeware for populations genetic analysis. http://www.ualberta.ca/;fyeh/index.htm (1999).

[22] Kalinowski ST, Taper ML, Marshall TC (2010). Revising how the computer program CERVUS accommodates genotyping error increases success in paternity assignment (vol 16, pg 1099, 2007). Mol. Ecol..

[23] Zhou Q (2020). Analysis of genetic diversity of ancient Ginkgo populations using SSR markers. Ind. Crops Prod..

[24] Hokanson SC, Lamboy WF, Szewc-McFadden AK, McFerson JR (2001). Microsatellite (SSR) variation in a collection of Malus (apple) species and hybrids. Euphytica.

[25] Kumar C (2019). Genetic diversity and population structure analysis of wild Malus genotypes including the crabapples (M. baccata (L.) Borkh. & M. sikkimensis (Wenzig) Koehne ex C. Schneider) collected from the Indian Himalayan region using microsatellite markers. Genet. Res. Crop Evol..

[26] Ai C, Xu LA, Lai HL, Huang MR, Wang ZR (2006). Genetic diversity and paternity analysis of a seed orchard in *Pinus massoniana*. Sci. Silvae Sin..

[27] Wu J-C, Zhang Y-P, Zheng Y-X, Peng X-M (2018). Pollen mediated gene flow in a small experimental population of Moringa oleifera Lam (Moringaceae). Ind. Crop. Prod..

[28] He TH, Ge S (2001). Mating system paternity anslysis and geneflow in plant populations. Acta Phytoecol. Sin. (Chin. J. Plant Ecol.).

[29] Gettys LA (2012). Genetic control of white flower color in scarlet rosemallow (Hibiscus coccineus Walter). J. Hered..

[30] Yan, D. F. *Studies on Inter-Species Hybridization of Camellia azalea and Genetic Trait Analysis of Its F*_*1*_* Hybrids*, Zhongkai University of Agriculture and Engineering (2013).

[31] Han F-Q (2015). Inheritance and InDel markers closely linked to petal color gene (cpc-1) in Brassica oleracea. Mol. Breed..

[32] Olsen RT, Ranney TG, Werner DJ (2006). Fertility and inheritance of variegated and purple foliage across a polyploid series in Hypericum androsaemum L. J. Am. Soc. Hortic. Sci..

[33] Roberts DJ, Werner DJ, Wadl PA, Trigiano RN (2015). Inheritance and allelism of morphological traits in eastern redbud (Cercis canadensis L). Hortic. Res..

[34] Hattemer HH, Steiner W, Kownatzki D (1990). Genetic markers in birch. Silvae Genet..

[35] Heinze B, Geburek T (1995). Searching for DNA markers linked to leaf colour in copper beech, Fagus sylvatica L. var atropunicea. Silvae Genetica.

[36] Luo YX (2011). Inheritance of leaf color and sequence-related amplified polymorphic (SRAP) molecular markers linked to the leaf color gene in Brassica juncea. Afr. J. Biotech..

[37] Huang C-H, Chu C-Y (2017). Inheritance of leaf and flower morphologies in Kalanchoe spp.. Euphytica.

[38] Crane MB, Lawrence WJC (1933). Genetical studies in cultivated apples. J. Genet..

[39] Cheng FS, Weeden NF, Brown SK (1996). Identification of co-dominant RAPD markers tightly linked to fruit skin color in apple. TAG. Theor. Appl. Genet. Theor. Angew. Genet..

[40] Costa F (2015). MetaQTL analysis provides a compendium of genomic loci controlling fruit quality traits in apple. Tree Genet. Genomes.

[41] Sheng BC, Yu ML (1993). Inheritance of fruit skin colour in apple progeny. J. Fruit Sci..

[42] Fisher JB, Honda H (1979). Branch geometry and effective leaf area: A study of terminalia-branching pattern. 2. Survey of real trees. Am. J. Bot..

[43] Chaparro JX, Werner DJ, O'Malley D, Sederoff RR (1994). Targeted mapping and linkage analysis of morphological isozyme, and RAPD markers in peach. TAG. Theor. Appl. Genet. Theor. Angew. Genet..

[44] Zhang J (2015). High-density genetic map construction and identification of a locus controlling weeping trait in an ornamental woody plant (Prunus mume Sieb. et Zucc). DNA Res..

[45] Dougherty L, Singh R, Brown S, Dardick C, Xu K (2018). Exploring DNA variant segregation types in pooled genome sequencing enables effective mapping of weeping trait in Malus. J Exp Bot.

[46] Shen P (2020). Genetic analysis of main flower characteristics in the F(1)generation derived from intraspecific hybridization between plumbago auriculata and plumbago auriculata f. alba. Sci. Hortic..

