# Supplementary material for: Pedigree reconstruction and genetic analysis of major ornamental characters of ornamental crabapple (Malus spp.) based on paternity analysis
Source: Sci Rep. 2022 Aug 18;12:14093. doi: 10.1038/s41598-022-18352-z (PMC9388634; doi:10.1038/s41598-022-18352-z)
Supplement: Supplementary file 1 — Supplementary Information 1. [file 41598_2022_18352_MOESM1_ESM.docx]

**Table S1** Ninety-one candidate parental materials

| No. | Cultivar | No. | Cultivar | No. | | Cultivar |
| --- | --- | --- | --- | --- | --- | --- |
| 1 | *M*. ‘floribunda’ | 32 | *M*. ‘Ballet’ | | 63 | *M*. ‘Sentinel’ |
| 2 | *M*. ‘Adirondack’ | 33 | *M*. ‘Radiant’ | | 64 | *M*. ‘Adams’ |
| 3 | *M*. ‘Pink Princess’ | 34 | *M*. ‘Sweet Sugartyme’ | | 65 | *M*. ‘Show Time’ |
| 4 | *M*. ‘Purple Pendula’ | 35 | *M*. ‘David’ | | 66 | M. ‘Eleyi’ |
| 5 | *M*. ‘Superstar’ | 36 | *M*. ‘Lancelot’ | | 67 | *M*. ‘Weeping Madonna’ |
| 6 | *M*. ‘Red Sentinel’ | 37 | *M*. ‘Lollipop’ | | 68 | *M*. ‘Louisa Contort’ |
| 7 | *M*. ‘Big Red’ | 38 | *M*. ‘Shelley’ | | 69 | *M*. ‘Almey’ |
| 8 | *M*. ‘Kelsey’ | 39 | *M*. ‘May’s Delight’ | | 70 | *M*. ‘Spring Sensation’ |
| 9 | *M*. ‘Indian Summer’ | 40 | *M*. ‘Indian magic’ | | 71 | *M*. ‘Snowdrift’ |
| 10 | *M*. ‘Hopa’ | 41 | *M*. ‘Lisa’ | | 72 | *M*. ‘Coralburst’ |
| 11 | *M*. ‘Lemoinei’ | 42 | *M*. ‘Pink ballet’ | | 73 | *M*. ‘Red Splendor’ |
| 12 | *M*. ‘Rainbow’ | 43 | *M* ‘Flame’ | | 74 | *M*. ‘Strawberry Jelly’ |
| 13 | *M*. ‘Perfect Purple’ | 44 | *M*. ‘Red Jade’ | | 75 | *M*. ‘Red Baron’ |
| 14 | *M*. ‘Molten Lava’ | 45 | *M*. ‘Dolgo’ | | 76 | *M* ‘Golden Hornet’ |
| 15 | *M*. ‘King Arthur’ | 46 | *M*. ‘Prairifire’ | | 77 | *M*. ‘Louisa’ |
| 16 | *M*. ‘Liset’ | 47 | *M*. ‘Makamik’ | | 78 | *M* ‘Neville Copeman’ |
| 17 | *M*. ‘Spring Snow’ | 48 | *M*. ‘Black Jade’ | | 79 | *M*. ‘Roudph’ |
| 18 | *M*. ‘Vans Eseltine’ | 49 | *M* ‘Selkirk’ | | 80 | *M*. ‘Sparkler’ |
| 19 | *M*. ‘Cinderella’ | 50 | *M*. ‘Evereste’ | | 81 | *M*. ‘Winter Red’ |
| 20 | *M*. ‘Winter Gold’ | 51 | *M*. ‘SugarTyme’ | | 82 | *M*. ‘Tina’ |
| 21 | *M*. ‘Ballet Red’ | 52 | *M*. ‘Guard’ | | 83 | *M*. ‘Bride’ |
| 22 | *M*. ‘Red Jewel’ | 53 | *M*. ‘Professor Sprenger’ | | 84 | *M*. ‘Roger’s Selection’ |
| 23 | *M*. ‘Mary Potter’ | 54 | *M*. ‘Irene’ | | 85 | *M*. ‘Cardinal’ |
| 24 | *M*. ‘Spring Glory’ | 55 | *M*. ‘Royal Raindrop’ | | 86 | *M*. ‘Gorgeous’ |
| 25 | *M*. ‘Darwin’ | 56 | *M*. ‘Centurion’ | | 87 | *M*. ‘Butterball’ |
| 26 | *M*. ‘Robinson’ | 57 | *M*. ‘Velvet Pillar’ | | 88 | *M*. ‘Royal Delicious’ |
| 27 | *M*. ‘Donald Wyman’ | 58 | *M*. ‘Purple Prince’ | | 89 | *M*. ‘Pink Spires’ |
| 28 | *M*. ‘Hydrangea’ | 59 | *M*. ‘Harvest Gold’ | | 90 | *M*. ‘Profusion’ |
| 29 | *M*. ‘John Downie’ | 60 | *M*. ‘Painted Scroll’ | | 91 | *M*. ‘Fairtail Gold’ |
| 30 | *M*. ‘White Cascade’ | 61 | *M*. ‘Yellow Jade’ | |  |  |
| 31 | *M.* ‘Zumi calocarpa’ | 62 | *M*. ‘Coccinella’ | |  |  |

**Table S2** Parents of 4 half-sib families (95% confidence)

| Offspring | Male parent | Offspring | Male parent | Offspring | Male parent | Offspring | Male parent |
| --- | --- | --- | --- | --- | --- | --- | --- |
| CH1 | 7 | DEW2 | 6 | HSB7 | 86 | XTT7 | 91 |
| CH2 | 6 | DEW3 | 6 | HSB8 | 30 | XTT8 | 67 |
| CH3 | 68 | DEW6 | 12 | HSB9 | 34 | XTT9 | 68 |
| CH6 | 19 | DEW7 | 6 | HSB10 | 66 | XTT10 | 6 |
| CH7 | 34 | DEW8 | 6 | HSB11 | 66 | XTT12 | 26 |
| CH8 | 6 | DEW9 | 6 | HSB14 | 87 | XTT14 | 86 |
| CH9 | 81 | DEW10 | 6 | HSB18 | 34 | XTT15 | 91 |
| CH10 | 6 | DEW11 | 81 | HSB19 | 61 | XTT17 | 68 |
| CH11 | 81 | DEW12 | 6 | HSB20 | 69 | XTT18 | 34 |
| CH13 | 81 | DEW14 | 6 | HSB23 | 34 | XTT19 | 67 |
| CH14 | 86 | DEW15 | 6 | HSB25 | 12 | XTT20 | 67 |
| CH15 | 89 | DEW17 | 6 | HSB26 | 7 | XTT21 | 6 |
| CH16 | 34 | DEW18 | 6 | HSB27 | 80 | XTT22 | 29 |
| CH18 | 68 | DEW19 | 6 | HSB28 | 34 | XTT23 | 91 |
| CH19 | 81 | DEW21 | 6 | HSB30 | 20 | XTT26 | 71 |
| CH20 | 6 | DEW22 | 6 | HSB32 | 87 | XTT27 | 91 |
| CH24 | 91 | DEW24 | 19 | HSB33 | 34 | XTT31 | 26 |
| CH25 | 67 | DEW27 | 6 | HSB34 | 29 | XTT32 | 6 |
| CH26 | 91 | DEW29 | 6 | HSB35 | 69 | XTT33 | 72 |
| CH27 | 62 | DEW30 | 6 | HSB38 | 69 | XTT35 | 52 |
| CH28 | 29 | DEW31 | 6 | HSB39 | 20 | XTT37 | 68 |
| CH29 | 81 | DEW34 | 6 | HSB41 | 91 | XTT38 | 19 |
| CH30 | 67 | DEW35 | 6 | HSB42 | 29 | XTT39 | 29 |
| CH31 | 6 | DEW39 | 6 | HSB44 | 26 | XTT40 | 71 |
| CH32 | 81 | DEW40 | 6 | HSB45 | 69 | XTT41 | 34 |
| CH33 | 6 | DEW42 | 6 | HSB48 | 30 | XTT42 | 91 |
| CH34 | 91 | DEW43 | 19 | HSB49 | 30 | XTT43 | 91 |
| CH36 | 81 | DEW47 | 6 | HSB51 | 34 | XTT44 | 67 |
| CH37 | 40 | DEW49 | 6 | HSB52 | 69 | XTT45 | 68 |
| CH38 | 46 | DEW50 | 6 | HSB53 | 34 | XTT47 | 35 |
| CH39 | 61 | DEW51 | 6 | HSB54 | 87 | XTT49 | 68 |
| CH40 | 81 | DEW54 | 6 | HSB55 | 91 | XTT50 | 38 |
| CH41 | 34 | DEW55 | 6 | HSB56 | 50 | XTT51 | 6 |
| CH42 | 6 | DEW56 | 6 | HSB57 | 51 | XTT53 | 26 |
| CH43 | 89 | DEW57 | 6 | HSB59 | 54 | XTT54 | 67 |
| CH45 | 6 | DEW58 | 81 | HSB60 | 58 | XTT55 | 29 |
| CH47 | 66 | DEW59 | 21 | HSB61 | 47 | XTT56 | 89 |
| CH48 | 7 | DEW60 | 6 | HSB63 | 91 | XTT57 | 41 |
| CH49 | 81 | DEW62 | 6 | HSB64 | 69 | XTT58 | 26 |
| CH51 | 81 | DEW63 | 6 | HSB65 | 29 | XTT59 | 6 |
| CH52 | 81 | DEW65 | 6 | HSB66 | 34 | XTT60 | 34 |
| CH54 | 34 | DEW66 | 58 | HSB67 | 29 | XTT61 | 68 |
| CH56 | 6 | DEW67 | 6 | HSB68 | 69 | XTT62 | 36 |
| CH59 | 6 | DEW68 | 22 | HSB69 | 66 | XTT63 | 67 |
| CH60 | 6 | DEW70 | 81 | HSB70 | 7 | XTT65 | 20 |
| CH61 | 81 | DEW72 | 6 | HSB73 | 30 | XTT66 | 31 |
| CH62 | 34 | DEW73 | 52 | HSB75 | 87 | XTT67 | 20 |
| CH63 | 81 | DEW74 | 6 | HSB76 | 34 | XTT70 | 91 |
| CH66 | 6 | DEW75 | 6 | HSB78 | 34 | XTT72 | 67 |
| CH68 | 67 | DEW76 | 6 | HSB79 | 34 | XTT73 | 26 |
| CH69 | 81 | DEW77 | 6 | HSB80 | 30 | XTT75 | 68 |
| CH70 | 69 | DEW78 | 6 | HSB82 | 12 | XTT76 | 91 |
| CH71 | 81 | DEW79 | 4 | HSB84 | 45 | XTT78 | 43 |
| CH72 | 34 | DEW80 | 81 | HSB85 | 20 | XTT79 | 19 |
| CH75 | 81 | DEW81 | 87 | HSB86 | 20 | XTT80 | 6 |
| CH78 | 81 | DEW83 | 6 | HSB89 | 29 | XTT81 | 6 |
| CH79 | 6 | DEW84 | 6 | HSB90 | 26 | XTT82 | 6 |
| CH80 | 81 | DEW85 | 6 | HSB91 | 9 | XTT83 | 6 |
| CH81 | 91 | DEW86 | 46 | HSB92 | 69 | XTT84 | 67 |
| CH82 | 81 | DEW89 | 6 | HSB93 | 68 | XTT85 | 86 |
| CH85 | 81 | DEW91 | 6 | HSB94 | 66 | XTT86 | 1 |
| CH86 | 81 | DEW92 | 6 | HSB95 | 69 | XTT88 | 68 |
| CH87 | 81 | DEW95 | 6 | HSB96 | 31 | XTT90 | 6 |
| CH89 | 81 | DEW96 | 22 | XTT1 | 36 | XTT94 | 67 |
| CH90 | 81 | HSB2 | 34 | XTT2 | 68 | XTT95 | 67 |
| CH91 | 19 | HSB3 | 72 | XTT3 | 67 | XTT96 | 43 |
| CH93 | 81 | HSB4 | 66 | XTT4 | 68 |  |  |
| CH94 | 34 | HSB5 | 22 | XTT5 | 20 |  |  |
| CH96 | 34 | HSB6 | 80 | XTT6 | 56 |  |  |

**Table S3** Phenotypic traits of recombinant siblings

| Combination | Offspring | Blooming period | Flower color | Leaf color | Leaf ship | Leaf hairiness surface | Tree habit | Fruit diameter | Fruit color | Calyx persistence | Glossiness of skin |
| --- | --- | --- | --- | --- | --- | --- | --- | --- | --- | --- | --- |
| *M*.‘Rainbow’× *M*. ‘Red Sentinel’ | CH2 | 2 | 1 | 1 | 5 | 2 | 2 | 3 | 2 | 1 | 2 |
|  | CH8 | 2 | 1 | 1 | 4 | 1 | 2 | 2 | 2 | 1 | 1 |
|  | CH10 | 2 | 1 | 1 | 4 | 1 | 2 | 3 | 2 | 1 | 1 |
|  | CH20 | 2 | 1 | 1 | 4 | 2 | 2 | 3 | 1 | 2 | 3 |
|  | CH31 | 2 | 1 | 1 | 4 | 1 | 1 | 2 | 2 | 2 | 1 |
|  | CH33 | 2 | 1 | 1 | 4 | 2 | 2 | 2 | 3 | 2 | 3 |
|  | CH42 | 2 | 1 | 1 | 4 | 2 | 2 | 3 | 2 | 1 | 1 |
|  | CH45 | 1 | 3 | 2 | 4 | 1 | 2 | 2 | 3 | 2 | 1 |
|  | CH56 | 2 | 1 | 1 | 4 | 2 | 2 | 3 | 2 | 1 | 2 |
|  | CH59 | 2 | 1 | 1 | 4 | 2 | 2 | 3 | 2 | 2 | 2 |
|  | CH60 | 1 | 2 | 1 | 5 | 2 | 3 | 2 | 3 | 2 | 2 |
|  | CH66 | 2 | 1 | 1 | 5 | 2 | 2 | 2 | 1 | 1 | 1 |
|  | CH79 | 2 | 1 | 1 | 5 | 2 | 2 | 3 | 2 | 1 | 3 |
| *M*. ‘Rainbow’× *M*. ‘Winter Red’ | CH9 | 2 | 1 | 1 | 4 | 1 | 2 | 3 | 1 | 1 | 1 |
|  | CH11 | 2 | 1 | 1 | 5 | 1 | 2 | 3 | 2 | 2 | 1 |
|  | CH13 | 2 | 1 | 1 | 5 | 0 | 2 | 2 | 3 | 1 | 1 |
|  | CH19 | 2 | 1 | 1 | 5 | 2 | 2 | 3 | 1 | 1 | 1 |
|  | CH29 | 2 | 2 | 1 | 4 | 2 | 1 | 2 | 1 | 1 | 1 |
|  | CH32 | 2 | 1 | 1 | 4 | 0 | 2 | 2 | 3 | 2 | 3 |
|  | CH36 | 1 | 2 | 1 | 5 | 1 | 2 | 2 | 1 | 1 | 3 |
|  | CH40 | 2 | 1 | 1 | 4 | 2 | 2 | 2 | 2 | 1 | 3 |
|  | CH49 | 2 | 1 | 1 | 5 | 2 | 2 | 2 | 2 | 2 | 2 |
|  | CH51 | 2 | 1 | 1 | 4 | 0 | 2 | 3 | 2 | 1 | 1 |
|  | CH52 | 1 | 1 | 1 | 4 | 2 | 2 | 2 | 2 | 2 | 2 |
|  | CH61 | 2 | 1 | 1 | 4 | 2 | 2 | 3 | 3 | 1 | 2 |
|  | CH63 | 2 | 1 | 1 | 4 | 2 | 3 | 2 | 3 | 1 | 1 |
|  | CH69 | 2 | 1 | 1 | 5 | 2 | 2 | 2 | 3 | 2 | 3 |
|  | CH71 | 2 | 1 | 1 | 4 | 0 | 2 | 2 | 3 | 2 | 1 |
|  | CH75 | 2 | 1 | 1 | 5 | 1 | 2 | 2 | 2 | 1 | 3 |
|  | CH78 | 1 | 1 | 1 | 4 | 2 | 2 | 2 | 1 | 2 | 1 |
|  | CH80 | 2 | 1 | 1 | 5 | 1 | 2 | 3 | 1 | 1 | 3 |
|  | CH82 | 1 | 1 | 1 | 4 | 2 | 2 | 2 | 3 | 1 | 1 |
|  | CH85 | 2 | 2 | 2 | 4 | 0 | 2 | 3 | 3 | 1 | 2 |
|  | CH86 | 2 | 1 | 1 | 5 | 2 | 2 | 2 | 2 | 2 | 1 |
|  | CH87 | 2 | 1 | 1 | 4 | 2 | 1 | 2 | 3 | 1 | 1 |
|  | CH89 | 2 | 1 | 1 | 4 | 2 | 2 | 3 | 2 | 2 | 3 |
|  | CH90 | 2 | 1 | 1 | 5 | 2 | 3 | 2 | 3 | 1 | 3 |
|  | CH93 | 2 | 1 | 1 | 4 | 2 | 2 | 2 | 3 | 2 | 1 |
| *M*. ‘Darwin’× *M*. ‘Red Sentinel’ | DEW2 | 1 | 1 | 1 | 4 | 1 | 2 | 2 | 1 | 1 | 1 |
|  | DEW3 | 2 | 2 | 1 | 4 | 1 | 3 | 3 | 2 | 1 | 1 |
|  | DEW7 | 1 | 2 | 1 | 4 | 2 | 2 | 3 | 2 | 2 | 1 |
|  | DEW8 | 2 | 1 | 1 | 4 | 1 | 2 | 3 | 3 | 1 | 2 |
|  | DEW9 | 2 | 2 | 1 | 4 | 2 | 2 | 3 | 3 | 2 | 2 |
|  | DEW10 | 1 | 2 | 1 | 4 | 1 | 1 | 3 | 3 | 1 | 1 |
|  | DEW12 | 2 | 1 | 1 | 4 | 2 | 2 | 2 | 2 | 2 | 1 |
|  | DEW14 | 2 | 1 | 1 | 4 | 2 | 1 | 3 | 1 | 1 | 1 |
|  | DEW15 | 2 | 2 | 1 | 4 | 2 | 2 | 3 | 2 | 1 | 2 |
|  | DEW17 | 2 | 1 | 1 | 4 | 1 | 2 | 3 | 2 | 1 | 2 |
|  | DEW18 | 2 | 1 | 1 | 4 | 2 | 2 | 3 | 2 | 2 | 1 |
|  | DEW19 | 2 | 2 | 1 | 4 | 2 | 2 | 2 | 3 | 2 | 2 |
|  | DEW21 | 1 | 1 | 2 | 4 | 2 | 2 | 2 | 3 | 1 | 1 |
|  | DEW22 | 2 | 2 | 3 | 4 | 2 | 2 | 3 | 3 | 1 | 1 |
|  | DEW27 | 2 | 2 | 1 | 4 | 0 | 2 | 3 | 2 | 1 | 1 |
|  | DEW29 | 2 | 2 | 1 | 4 | 2 | 2 | 2 | 3 | 2 | 1 |
|  | DEW30 | 2 | 1 | 1 | 4 | 2 | 2 | 3 | 2 | 2 | 1 |
|  | DEW31 | 1 | 2 | 1 | 4 | 1 | 3 | 3 | 3 | 2 | 1 |
|  | DEW34 | 2 | 1 | 1 | 5 | 2 | 2 | 3 | 1 | 2 | 1 |
|  | DEW35 | 1 | 2 | 3 | 4 | 2 | 3 | 1 | 3 | 1 | 2 |
|  | DEW39 | 2 | 2 | 3 | 4 | 2 | 3 | 2 | 2 | 1 | 1 |
|  | DEW40 | 1 | 1 | 1 | 4 | 1 | 2 | 3 | 3 | 2 | 1 |
|  | DEW42 | 1 | 1 | 1 | 4 | 2 | 2 | 3 | 3 | 1 | 1 |
|  | DEW47 | 2 | 1 | 1 | 4 | 2 | 4 | 2 | 2 | 1 | 1 |
|  | DEW49 | 1 | 1 | 1 | 4 | 1 | 2 | 2 | 2 | 1 | 2 |
|  | DEW50 | 2 | 1 | 1 | 4 | 1 | 2 | 3 | 1 | 1 | 3 |
|  | DEW51 | 2 | 2 | 2 | 4 | 0 | 2 | 2 | 3 | 2 | 1 |
|  | DEW54 | 1 | 2 | 1 | 5 | 1 | 2 | 3 | 3 | 1 | 1 |
|  | DEW55 | 1 | 2 | 3 | 4 | 2 | 2 | 3 | 3 | 2 | 2 |
|  | DEW56 | 1 | 1 | 1 | 4 | 1 | 2 | 1 | 2 | 1 | 1 |
|  | DEW57 | 2 | 2 | 1 | 4 | 1 | 3 | 3 | 2 | 2 | 1 |
|  | DEW60 | 1 | 1 | 1 | 4 | 2 | 2 | 3 | 2 | 1 | 1 |
|  | DEW62 | 2 | 2 | 1 | 2 | 2 | 2 | 3 | 1 | 2 | 1 |
|  | DEW63 | 2 | 1 | 1 | 5 | 2 | 2 | 3 | 2 | 1 | 1 |
|  | DEW65 | 2 | 2 | 2 | 4 | 2 | 2 | 3 | 3 | 2 | 1 |
|  | DEW67 | 2 | 2 | 1 | 4 | 1 | 3 | 3 | 3 | 1 | 1 |
|  | DEW72 | 2 | 1 | 1 | 4 | 1 | 2 | 3 | 2 | 1 | 1 |
|  | DEW74 | 2 | 1 | 1 | 4 | 1 | 2 | 3 | 2 | 2 | 1 |
|  | DEW75 | 2 | 1 | 1 | 4 | 1 | 2 | 2 | 2 | 2 | 3 |
|  | DEW76 | 2 | 2 | 1 | 5 | 2 | 2 | 3 | 3 | 1 | 1 |
|  | DEW77 | 2 | 2 | 1 | 4 | 1 | 2 | 1 | 3 | 1 | 1 |
|  | DEW78 | 2 | 2 | 1 | 5 | 2 | 2 | 2 | 3 | 2 | 1 |
|  | DEW83 | 1 | 1 | 2 | 5 | 0 | 2 | 3 | 2 | 1 | 1 |
|  | DEW84 | 2 | 2 | 3 | 4 | 1 | 3 | 3 | 3 | 2 | 2 |
|  | DEW85 | 2 | 1 | 1 | 2 | 2 | 2 | 3 | 2 | 2 | 1 |
|  | DEW89 | 1 | 1 | 1 | 4 | 1 | 2 | 3 | 2 | 1 | 1 |
|  | DEW91 | 1 | 2 | 3 | 4 | 2 | 1 | 2 | 3 | 2 | 1 |
|  | DEW92 | 2 | 1 | 1 | 4 | 1 | 2 | 3 | 3 | 1 | 2 |
|  | DEW95 | 2 | 1 | 1 | 5 | 2 | 2 | 3 | 2 | 2 | 1 |
| *M*. ‘Red Sentinel’× *M*. ‘Sweet Sugartyme’ | HSB2 | 2 | 1 | 1 | 4 | 2 | 2 | 3 | 3 | 1 | 1 |
|  | HSB9 | 2 | 1 | 1 | 4 | 2 | 2 | 2 | 2 | 2 | 2 |
|  | HSB18 | 2 | 1 | 1 | 4 | 1 | 2 | 3 | 2 | 1 | 2 |
|  | HSB23 | 2 | 1 | 1 | 4 | 1 | 2 | 3 | 1 | 1 | 2 |
|  | HSB28 | 2 | 1 | 1 | 4 | 1 | 2 | 3 | 1 | 1 | 2 |
|  | HSB33 | 2 | 1 | 1 | 4 | 1 | 2 | 3 | 1 | 1 | 2 |
|  | HSB51 | 2 | 1 | 1 | 4 | 1 | 2 | 2 | 2 | 1 | 3 |
|  | HSB53 | 1 | 1 | 1 | 5 | 1 | 1 | 2 | 3 | 1 | 2 |
|  | HSB66 | 2 | 1 | 1 | 4 | 1 | 2 | 2 | 1 | 1 | 3 |
|  | HSB76 | 2 | 1 | 1 | 4 | 1 | 2 | 2 | 1 | 1 | 2 |
|  | HSB78 | 2 | 1 | 1 | 4 | 1 | 3 | 3 | 2 | 2 | 3 |
|  | HSB79 | 2 | 1 | 1 | 4 | 1 | 2 | 3 | 2 | 1 | 2 |
| *M*. ‘Sweet Sugartyme’× *M*. ‘Red Sentinel’ | XTT10 | 1 | 1 | 1 | 4 | 1 | 3 | 3 | 3 | 1 | 2 |
|  | XTT21 | 2 | 1 | 1 | 4 | 1 | 2 | 3 | 3 | 1 | 1 |
|  | XTT32 | 2 | 1 | 1 | 4 | 2 | 2 | 3 | 3 | 1 | 1 |
|  | XTT51 | 1 | 1 | 1 | 4 | 1 | 2 | 3 | 2 | 1 | 2 |
|  | XTT59 | 2 | 1 | 1 | 5 | 2 | 1 | 3 | 3 | 2 | 1 |
|  | XTT80 | 2 | 1 | 1 | 4 | 1 | 2 | 3 | 2 | 1 | 2 |
|  | XTT81 | 2 | 1 | 1 | 4 | 2 | 2 | 3 | 2 | 1 | 1 |
|  | XTT82 | 2 | 1 | 1 | 5 | 1 | 1 | 3 | 3 | 1 | 1 |
|  | XTT83 | 2 | 1 | 1 | 4 | 1 | 2 | 3 | 2 | 2 | 2 |
|  | XTT90 | 2 | 1 | 1 | 4 | 2 | 2 | 3 | 3 | 1 | 2 |
| *M*. ‘Sweet Sugartyme’× *M*. ‘Weeping Madonna’ | XTT3 | 2 | 1 | 1 | 4 | 1 | 2 | 3 | 3 | 1 | 3 |
|  | XTT8 | 2 | 1 | 1 | 4 | 1 | 3 | 3 | 2 | 1 | 1 |
|  | XTT19 | 2 | 1 | 1 | 4 | 2 | 2 | 3 | 3 | 1 | 3 |
|  | XTT20 | 2 | 1 | 1 | 4 | 2 | 2 | 3 | 2 | 1 | 3 |
|  | XTT44 | 2 | 1 | 1 | 4 | 2 | 2 | 3 | 3 | 1 | 2 |
|  | XTT54 | 2 | 1 | 1 | 4 | 1 | 1 | 3 | 3 | 2 | 3 |
|  | XTT63 | 2 | 1 | 1 | 4 | 0 | 2 | 3 | 2 | 2 | 2 |
|  | XTT72 | 2 | 1 | 1 | 4 | 1 | 4 | 3 | 3 | 1 | 1 |
|  | XTT84 | 2 | 1 | 1 | 2 | 1 | 2 | 3 | 3 | 2 | 2 |
|  | XTT94 | 2 | 1 | 1 | 4 | 2 | 2 | 3 | 3 | 1 | 3 |
|  | XTT95 | 2 | 1 | 1 | 4 | 1 | 2 | 3 | 2 | 1 | 3 |
| *M*. ‘Sweet Sugartyme’× *M*. ‘Louisa Contort’ | XTT2 | 1 | 1 | 1 | 4 | 1 | 2 | 3 | 1 | 1 | 3 |
|  | XTT4 | 2 | 1 | 1 | 4 | 1 | 2 | 3 | 2 | 2 | 3 |
|  | XTT9 | 2 | 1 | 1 | 4 | 1 | 3 | 3 | 3 | 2 | 2 |
|  | XTT17 | 2 | 1 | 1 | 4 | 2 | 4 | 3 | 3 | 1 | 1 |
|  | XTT37 | 2 | 1 | 1 | 4 | 1 | 2 | 3 | 3 | 1 | 3 |
|  | XTT45 | 2 | 1 | 1 | 4 | 1 | 2 | 3 | 3 | 1 | 2 |
|  | XTT49 | 2 | 1 | 1 | 4 | 2 | 2 | 3 | 3 | 1 | 1 |
|  | XTT61 | 2 | 1 | 1 | 4 | 1 | 3 | 3 | 3 | 1 | 1 |
|  | XTT75 | 2 | 1 | 1 | 4 | 1 | 2 | 3 | 3 | 1 | 3 |
|  | XTT88 | 2 | 1 | 1 | 4 | 2 | 2 | 3 | 3 | 1 | 1 |
